# Case of Incarcerated Scrotal Hernia of the Omentum, in Which from the Absence of Urgent Symptoms, No Operation Was Required

**Published:** 1826-07

**Authors:** 

**Affiliations:** St. George's Hospital.


### CASES OF STRANGULATED HERNIA, *not requiring Operation.*

*Case of incarcerated Scrotal Hernia of the Omentum, in which, from the Absence of urgent Symptoms, no Operation was required.* Treated by Mr. Brodie, at St. George's Hospital.

May 15th, 1826.—Stephen King, ætat. about thirty; admitted at seven o'clock this morning, with a scrotal hernia on the right side, which had come down on Saturday (the day before yesterday), in consequence of his having broken his truss, and been irreducible since that time, although he had always been able to return it for the last fifteen years, during which period he has laboured under the disease. Took some opening medicine yesterday (Sunday), which operated very many times (as he expresses it,) both upwards and downwards: he likewise had a stool early this morning. The tumor is nearly of the size of a man's fist, unattended with pain or any unpleasant symptom, except its being irreducible.

As the attempts to reduce it proved unsuccessful, he was advised to remain, and use the bath, &c.; feeling no inconvenience, however, and having urgent business, he refused to stop then, but promised to return in a few hours.

Took Ol. Ricini, ℥i.

Returned at three P.M., having had three liquid motions from the oil, and the tumor being exactly in the same state, and still unattended by pain. Was put into the warm bath, bled, and took Tinct. Opii, gtt. xl. Being then faint, the taxis was employed, and kept up for twenty minutes, during which time something appeared to pass up, and the tumor became rather smaller; but still its reduction was not accomplished.

At a quarter-past seven, he was again put into the bath, and remained there till Mr. Brodie's arrival, at a quarter-past eight, at which time he was very faint. The tumor being then examined, was found distinctly to consist of omentum, having several hard knobs or masses in it; but no distinct feeling of intestine was perceptible either to Mr. Brodie or Mr. Jeffreys. Although the hernia remained unreduced, and was rather painful, still, being unattended by any thing like urgent symptoms, the operation was not thought necessary.

Four P.M.—VS. ad ℥xviij.

Eleven P.M.—VS. ad ℥xij.

℞. Magnesiæ Sulphat. ʒi. ex Haust. Salin. ℥iss. 3tis horis.

16th, seven A.M.—Bowels not opened; in other respects as before.

Ol. Ricini, ℥i.

Three P.M.—VS. ad ℥x. During the evening and following night, he had several evacuations.

17th.—He is free from all bad symptoms.

24th.—Discharged, free from complaint; the tumor remaining unaltered, and being supported by a suspensory bandage. He has since applied a truss.

---

The following case is analogous to the preceding:*

Mr. ——, aged fifty-one, has had inguinal hernia of the right side about seventeen years, which was originally brought on by running violently for six miles after a stage-coach. The first symptom of its existence was a swelling in the scrotum, with dull aching pain. This was allowed to go on for a fortnight before any thing was done: the hernia was then returned, and has been kept up ever since by means of a truss. It has always been reducible by the patient placing himself in the horizontal position, raising his lower extremities, and using gentle and steady pressure with his hand. But, on the 25th of February, 1826, it came down, in consequence of the patient having broken his truss, and at the same time taken unusual exercise, (having walked twelve miles.) In addition to the want of pressure from without, there was increased pressure from within, as Mr. —— had become eight inches larger round the abdomen within the last twelvemonths. On endeavouring to reduce it as usual, he did not succeed, and now felt at one part of the tumor a lump about the size of a testis.

* This case occurred in the *private* practice of an eminent surgeon, on which account we are not at liberty to give the name: our readers may rely upon its accuracy.

February 26th, (Sunday.)—Went to church, but, when he returned, he felt so ill as to go to bed: his bowels acted, and he lived as usual.

27th.—Went out a little way, but was obliged to return: felt still worse. Bowels open.

28th.—Still worse; not able to stand.

March 1st.—(A surgeon was called in.) The tumor is red, inflamed, and very painful when pressed. There is a large hard knot at the inferior part of it, which, if squeezed, produces sickness and pain, similar to what is experienced on squeezing the testis. When in the horizontal posture, and quiet, he feels no constitutional disturbance whatever. The pulse and tongue are natural, but the bowels have not been open to-day. If he stands up, sickness and faintness are the immediate consequence. It is conjectured that the rupture consists of omentum, which adheres, and is doubled or coiled layer after layer upon itself; thus accounting for the hard substance at the inferior part of the tumor.

A brisk dose of jalap, &c. to be taken immediately.

Evening.—The bowels have been freely opened.

2d.—Not worse. Pain on pressure in the course of the inguinal canal.

Leeches to be applied to the tumor. Continue the purgative medicine occasionally.

3d.—Better. Less pain on pressure.

Repeat the leeches; keep the bowels acting.

From this time he gradually got well, the leeches having been again repeated, and a lotion applied. At first he wore a suspensory belt, but was soon able to wear his truss as formerly. The knotted omentum is not gone, but is less. His general health is now as good as ever.

